# Alterations of BDNF and trkB mRNA Expression in the 6-Hydroxydopamine-Induced Model of Preclinical Stages of Parkinson’s Disease: An Influence of Chronic Pramipexole in Rats

**DOI:** 10.1371/journal.pone.0117698

**Published:** 2015-03-04

**Authors:** Klemencja Berghauzen-Maciejewska, Jadwiga Wardas, Barbara Kosmowska, Urszula Głowacka, Katarzyna Kuter, Krystyna Ossowska

**Affiliations:** Department of Neuro-Psychopharmacology, Institute of Pharmacology, Polish Academy of Sciences, 12 Smętna St., 31-343, Kraków, Poland; Universidade de São Paulo, BRAZIL

## Abstract

Our recent study has indicated that a moderate lesion of the mesostriatal and mesolimbic pathways in rats, modelling preclinical stages of Parkinson’s disease, induces a depressive-like behaviour which is reversed by chronic treatment with pramipexole. The purpose of the present study was to examine the role of brain derived neurotrophic factor (BDNF) signalling in the aforementioned model of depression. Therefore, we investigated the influence of 6-hydoxydopamine (6-OHDA) administration into the ventral region of the caudate-putamen on mRNA levels of BDNF and tropomyosin-related kinase B (trkB) receptor. The BDNF and trkB mRNA levels were determined in the nigrostriatal and limbic structures by *in situ* hybridization 2 weeks after the operation. Pramipexole (1 mg/kg sc twice a day) and imipramine (10 mg/kg ip once a day) were injected for 2 weeks. The lesion lowered the BDNF and trkB mRNA levels in the hippocampus [CA1, CA3 and dentate gyrus (DG)] and amygdala (basolateral/lateral) as well as the BDNF mRNA content in the habenula (medial/lateral). The lesion did not influence BDNF and trkB expression in the caudate-putamen, substantia nigra, nucleus accumbens (shell and core) and ventral tegmental area (VTA). Chronic imipramine reversed the lesion-induced decreases in BDNF mRNA in the DG. Chronic pramipexole increased BDNF mRNA, but decreased trkB mRNA in the VTA in lesioned rats. Furthermore, it reduced BDNF and trkB mRNA expression in the shell and core of the nucleus accumbens, BDNF mRNA in the amygdala and trkB mRNA in the caudate-putamen in these animals. The present study indicates that both the 6-OHDA-induced dopaminergic lesion and chronic pramipexole influence BDNF signalling in limbic structures, which may be related to their pro-depressive and antidepressant activity in rats, respectively.

## Introduction

Primary motor symptoms of Parkinson’s disease (PD), such as bradykinesia, muscle rigidity and tremor result from massive degeneration of dopaminergic neurons of the nigrostriatal pathway which leads to a dramatic decrease in the dopamine level in the putamen and caudate nucleus. The clinical phase of PD is preceded by the preclinical period where depression is a frequent comorbid disturbance. The mechanisms underlying depression in PD are unclear. It is hypothesized that changes in brain structure and neurotransmitter signalling pathways, particularly abnormalities in dopaminergic, noradrenergic and serotoninergic projections may substantially contribute to its development [1].

Emerging pieces of evidence suggest that alterations of brain-derived neurotrophic factor (BDNF) expression play an important role in depression. BDNF belongs to a family of related proteins called neurotrophins. Acting through the tropomyosin-related kinase B (trkB) receptor, it transduces intracellular signalling events that are critical for axonal growth, neuronal survival and plasticity throughout the whole lifespan [2–5].

The neurotrophic hypothesis of depression postulates that neurotrophin deficiency contributes to the pathology (atrophy) of the hippocampus and supplementing these deficits by antidepressant drugs reverses the symptoms of the disease [2,3,6,7]. Brain imaging studies of depressed patients demonstrated decreased volumes of several brain structures, including the hippocampus, prefrontal cortex and amygdala, i.e. the regions linked to altered mood, anxiety and cognition [3,5,8,9].

Moreover, postmortem studies in depressed patients showed reduced expression of BDNF mRNA and protein levels in the hippocampus and prefrontal cortex as well as in the serum and plasma of patients with depression [10–13]. However, an opposite change, namely an increase in BDNF mRNA expression in the nucleus accumbens (a structure which is a part of the so-called ventral striatum) of depressed patients was observed by Krishnan and co-workers [14]. The above alterations were reversed by administration of different classes of antidepressant drugs [10,15–19], which paralleled the time course of their clinical response.

The investigations in animal models of stress and depression showed a decrease in BDNF mRNA and protein levels in the hippocampus, prefrontal cortex, frontal cortex and amygdala [20–26], and an increase in the nucleus accumbens and ventral tegmental area (VTA) [27,28], which could indicate a distinct role of BDNF in different brain structures.

The role of BDNF in depression in PD is unknown. Post-mortem studies of PD patients indicated a reduction of BDNF mRNA and protein levels in the substantia nigra pars compacta [29], caudate nucleus and putamen [30,31]. The studies conducted in animal models of PD were focused only on BDNF alterations in the nigro-striatal pathway. However, their results were unclear and varied depending on the degree of damage of the dopaminergic structures, and the type and the doses of the toxins used in the experiment [32–35].

Clinical studies indicate that the dopamine D3/D2 receptor agonist pramipexole is the most effective compound in the treatment of depression in PD [36]. The action of the classical tricyclic antidepressant drug imipramine in PD patients is controversial. On the one hand, its antidepressant effects in PD patients have been reported, but on the other, this drug causes a number of side effects and may even worsen the condition of patients [37].

Our recent studies [38,39] showed that a moderate dopaminergic lesion modelling a preclinical stage of PD induced by bilateral injections of 6-hydroxydopamine (6-OHDA) into the ventral region of the caudate-putamen evoked depressive-like behaviour in rats, namely an increase in immobility time in the forced swimming test. Furthermore, in line with its clinical antidepressant efficiency chronic pramipexole inhibited this behaviour while imipramine was ineffective [39]. Similarly, chronic pramipexole administration in rats was found to reverse a reduction of operant behaviour (a model of apathy) induced by 6-OHDA injected into the substantia nigra [40]. Imipramine was shown to shorten immobility time in naive animals [39], however, at the same time it induced excessive sedative effect in the lesioned rats which could mask its proper antidepressant action [39].

The purpose of the present study was to examine the role of BDNF signalling in the aforementioned animal model of depression in the preclinical phase of PD and the antidepressant-like effect of chronic pramipexole [39]. The BDNF and trkB mRNA expression in limbic and nigro-striatal structures was analysed using *in situ* hybridization. Imipramine was used as a reference compound. This drug has been repeatedly found to increase BDNF level in the hippocampus in animals, which has been suggested to contribute to its antidepressant properties [6,22,41–44].

## Materials and Methods

### Animals

The experiments were carried out in compliance with the Animal Experiments Bill of January 21, 2005; (published in Journal of Laws no. 33/2005 item 289, Poland), and according to the EC Directive 86/609/EEC on the protection of animals used for scientific purposes. They received also an approval of the Local Ethics Committee at the Institute of Pharmacology, Polish Academy of Sciences (Permit Number: 709, issued: January 28, 2010. All efforts were made to minimize the number and suffering of animals used.

Male Wistar rats (Charles River, Hannover, Germany), 9–11.5 weeks of age, weighing 250–300 g prior to experiments were kept under a 12/12 h light/dark cycle (the light on from 7 am to 7 pm) with free access to food and water. All experiments were carried out during the light period. Behaviours of these rats were analysed previously in the forced swimming test and in actometers [39].

### Operations

Under the pentobarbital anaesthesia (Vetbutal, Biowet, Poland; 25 mg/kg, *ip*) the animals were fixed into the stereotaxic instrument (Stoelting, USA) and injected bilaterally with 6-OHDA HBr [Sigma, Aldrich, 15 μg (free base) /2.5 μl per side, dissolved in 0.2% ascorbic acid] into the ventrolateral region of the caudate-putamen (AP: 1.2 mm, L: ± 3.1 mm, V: 6.8–7.0 mm from bregma according to Paxinos and Watson atlas [45]. Sham-operated rats which received 2.5 μl of 0.2% ascorbic acid bilaterally into the above region served as controls in all experiments. The injection cannulae were left in place for 60 s to enable full absorption of the solution. In order to spare noradrenergic terminals, desipramine (Sigma, Aldrich) was administered in a dose of 15 mg/2ml/kg ip 30 min before 6-OHDA injections. To avoid infections, the rats received an antibiotic (Lincospectin, Pharmacia, Belgium) 24 h before the operation, on the day of operation and 24 h afterwards.

### Drugs

Drugs or physiological saline were injected repeatedly for 2 weeks. Imipramine hydrochloride (Sigma, Aldrich) was dissolved in redistilled water and administered at a dose of 10 mg/kg ip, once a day. Pramipexole dihydrochloride (Abcam Ascent) was dissolved in physiological saline and administered at a dose of 1 mg/kg sc twice a day, except for the last day when it was injected only once. The first drug injection was performed one day after the operation. Control animals received physiological saline instead of drugs.

### *In situ* hybridization of BDNF and trkB receptor mRNAs

Rats were killed by decapitation 24 h after the last drug injection (4 h 30 min after examination of locomotor activity in actometers and 30 min after the forced swimming test [39]. The right side was used for HPLC analyses of levels of dopamine and its metabolites, as a measure of the lesion extent [39]. The left side of the brain was frozen in cold heptane on dry ice and stored at -80°C. Coronal sections (10 μm) of the nucleus accumbens, caudate-putamen, hippocampus, amygdala, habenula, substantia nigra and VTA were cut using a cryostat microtome at -20°C.

The sections were then thaw-mounted on gelatin-coated microscopic slides, postfixed in 4% paraformaldehyde for 10 min, dehydrated in an ascending series of alcohols, delipidated in chloroform, rehydrated in a descending series of alcohols, air-dried, and processed for *in situ* hybridization.

A 46–48-mer synthetic oligonucleotide probes were labelled with [35S]dATP (1000 Ci/mmol, Hartmann Analytic, Germany) in a 3’-tailing reaction using the terminal deoxynucleotidyl transferase enzyme (Fermentas, Lithuania) at 37°C for 30–45 min, to obtain a specific activity of about 5–6 x 10^5^ cpm/μl. Radioactive probe was purified using a phenol:chloroform standard protocol. The probes were complementary to bases: 1075–1123 and 982–1030 of the BDNF gene mRNA (GenBank accession number gi:120564500) and 2571–2617 of the trkB gene mRNA (GenBank accession number gi:207473). Sequence homology with other genes was verified using a GenBank BLAST program. Synthesis was performed at the DNA Sequencing and Oligonucleotide Synthesis Laboratory, Institute of Biochemistry and Biophysics, PAS (Warsaw, Poland).

The tissue sections were incubated in a hybridization buffer [50% formamide, 10% dextran sulfate, 0.25 mg/ml tRNA, 0.5 mg/ml salmon shared and denatured sperm DNA, 1x Denhardt solution, 4x saline-sodium citrate (SSC)] with the radiolabelled oligonucleotide (4–5x 10^5^ cpm per tissue section) for 20 h at 37°C in humidified chambers. All solutions were prepared in deionized 0.1% DEPC-treated water. After washing (5 min in 1x SSC RT, 3x 20 min in 2x SSC at 42°C, 1x 15 min in 1x SSC at a room temperature), the sections were dehydrated in ethanol, air-dried and exposed to a Kodak Bio-Max MR film (Sigma, Aldrich) for 4 weeks at 4°C. After exposure, the film was developed with a Dectol developer (Kodak, Sigma Aldrich), fixed with a GBX fixer/replenisher (Kodak, Sigma, Aldrich) and dried. To assess the specificity of probes the hybridization in some tissue sections was carried out in the presence of a 100-fold excess of the unlabeled probes, which eliminated the signal with the cDNA probes.

Signal density [the mean optical density (Q) minus background (B) per area unit (pixel^2^) of the region of interest] was measured in the above structures in the scanned images using Multi Gauge 3.0 program (Fujifilm Europe, GmbH, Poland). The mRNA expression was estimated in the nucleus accumbens (core and shell) at levels from the AP = 2.04 to 1.68 mm, caudate putamen at levels from the AP = 0.48 to -0.24 mm, hippocampus (CA1, CA3 and dentate gyrus (DG)) at levels from the AP = -2.28 to -3.24 mm, amygdala (basolateral + lateral amygdaloid nuclei) and habenula (medial + lateral) at levels from the AP = -2.76 to -3.48 mm, substantia nigra (pars compacta + reticulata) and VTA at levels from the AP = -4.68 to -6.60 mm from bregma according to the stereotaxic atlas of Paxinos and Watson [45].

### Statistics

A two-way ANOVA was used for statistical evaluation of *in situ* hybridization data, separately for rats treated with pramipexole and imipramine. The following pairs of factors were used: “lesion and pramipexole” or “lesion and imipramine”. When the effect of the drug (pramipexole or imipramine), the effect of the lesion or the lesion x drug interaction were significant (or of borderline statistical significance), the LSD post-hoc test was used for individual comparisons. P values of less than or equal to 0.05 were considered to indicate statistical significance. All statistical calculations were done using STATISTICA 7.0 Software (Statsoft, Inc. USA).

## Results

### The influence of the 6-OHDA-induced lesion on the BDNF and trkB mRNA expression in brain structures

**Fig 1:**
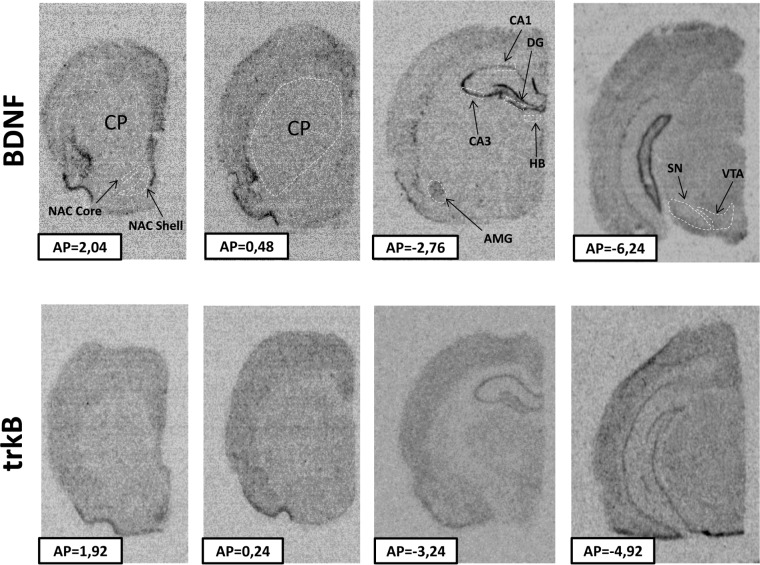
Representative autoradiograms showing BDNF and trkB mRNAs expression in frontal sections of the brain. Regions of interest are outlined. AMG—amygdala, CP—caudate-putamen, DG—dentate gyrus, HB—habenula, NAC—nucleus accumbens, SN—substantia nigra, VTA—ventral tegmental area. AP—anterior-posterior levels according to Paxinos and Watson [45].

**Fig 2:**
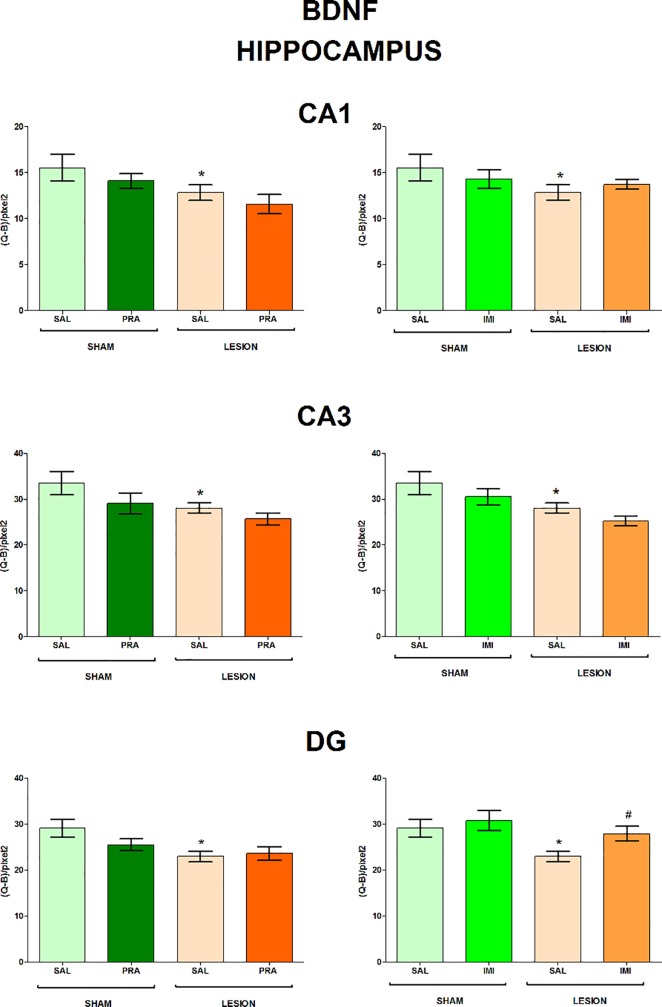
The influence of 6-OHDA, pramipexole and imipramine on BDNF mRNA in the hippocampus. The results are expressed as the mean ± S.E.M. LESION—lesioned rats, IMI—imipramine, PRA—pramipexole, Q-B/pixel2—the mean optical density-background per area units, SAL—saline, SHAM—sham-operated rats, n = 7–10, differences between SHAM-SAL vs. other groups are indicated by *p≤0.05, and differences between LESION-SAL vs. other groups by #p≤0.05). For further explanations see Fig. 1.

**Fig 3:**
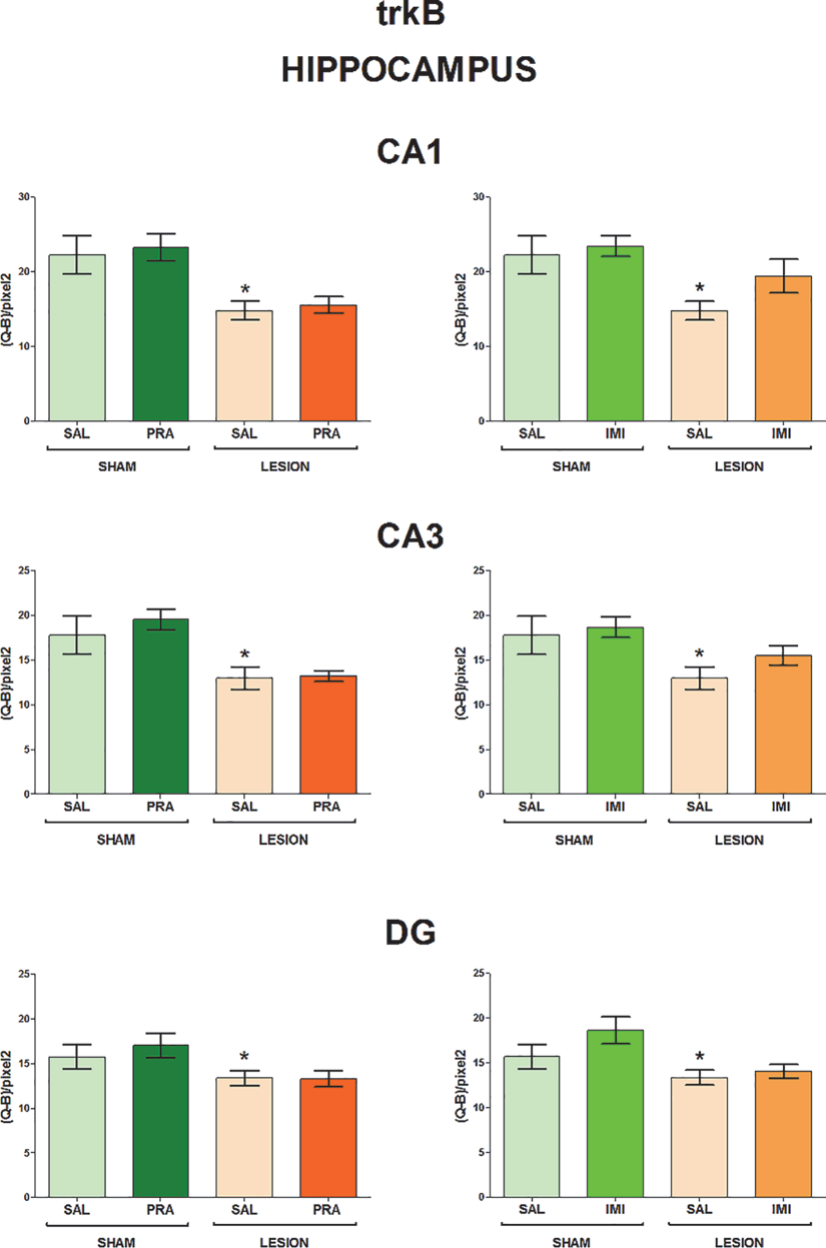
The influence of 6-OHDA, pramipexole and imipramine on trkB mRNA in the hippocampus. n = 9–10. For further explanations see Figs. 1 and 2.

**Fig 4:**
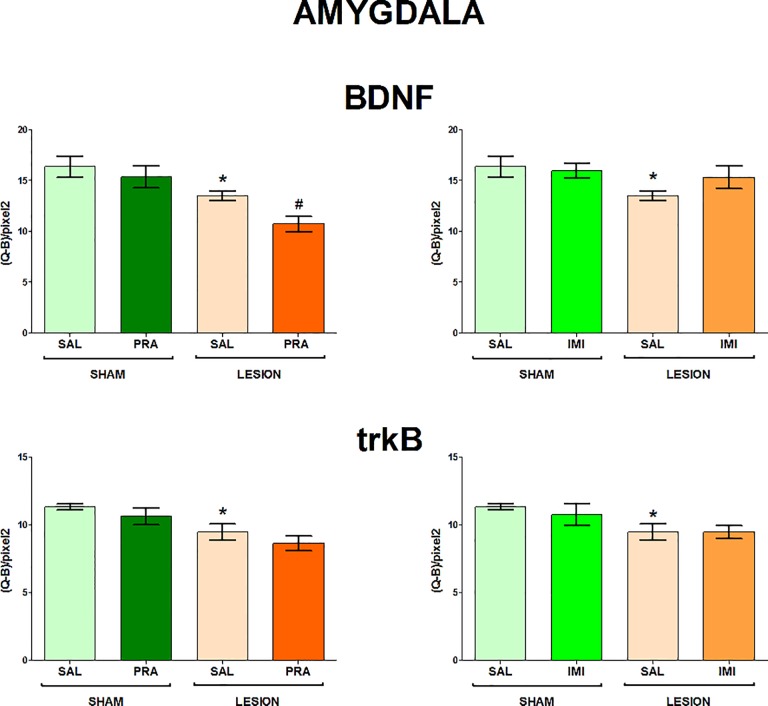
The influence 6-OHDA, pramipexole and imipramine on BDNF and trkB mRNAs in the amygdala. n = 8–10. For further explanations see Figs. 1 and 2.

**Fig 5:**
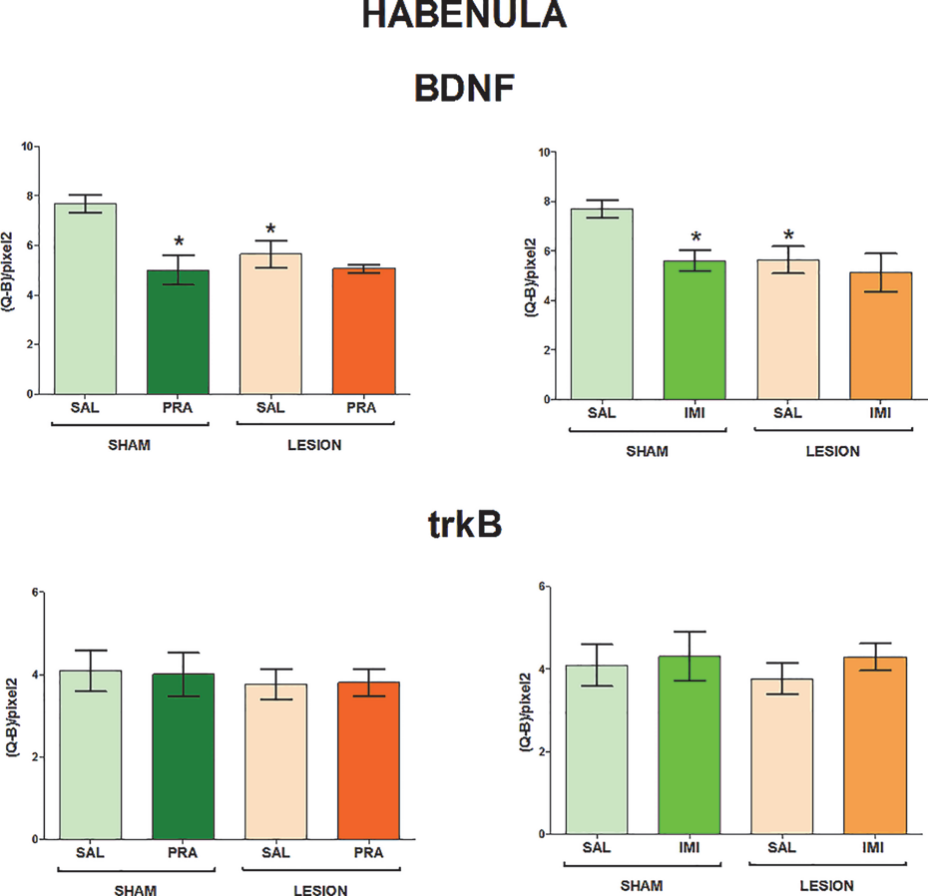
The influence of 6-OHDA, pramipexole and imipramine on BDNF and trkB mRNAs in the habenula. n = 8–10. For further explanations see Figs. 1 and 2.

**Fig 6:**
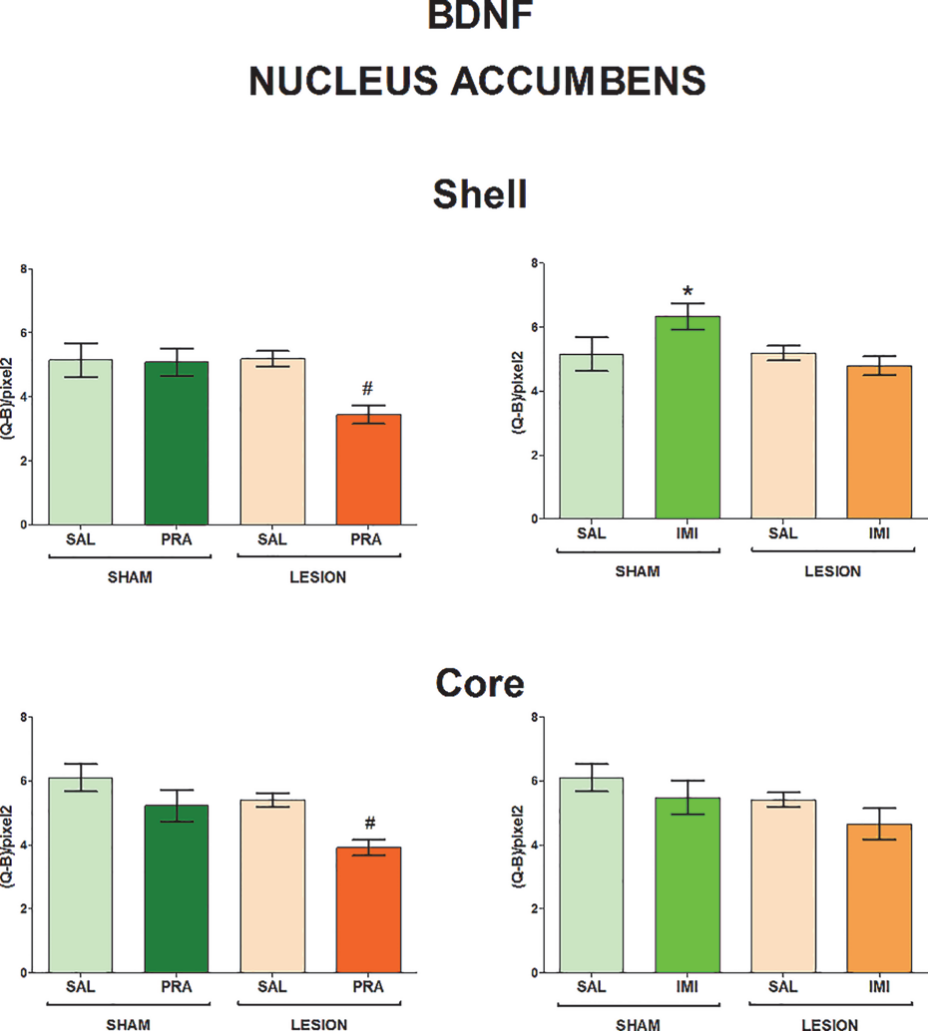
The influence of 6-OHDA, pramipexole and imipramine on BDNF mRNA in the nucleus accumbens. **n = 9–10.** For further explanations see Figs. 1 and 2.

**Fig 7:**
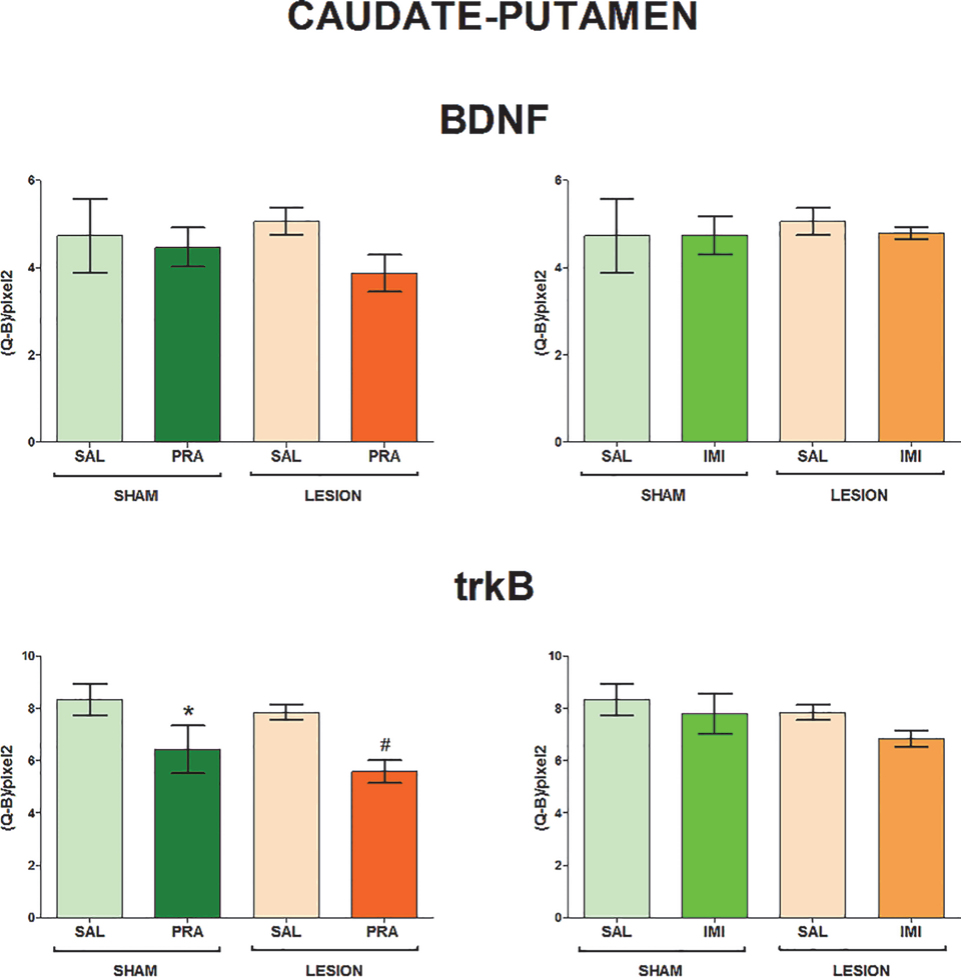
The influence of 6-OHDA, pramipexole and imipramine on BDNF and trkB mRNAs in the caudate-putamen. n = 9–10. For further explanations see Figs. 1 and 2.

**Fig 8:**
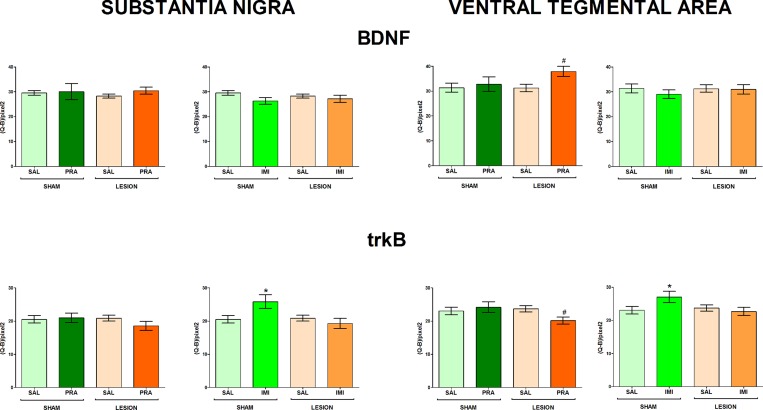
The influence of 6-OHDA, pramipexole and imipramine on BDNF and trkB mRNAs in mesencephalic structures. n = 5–10 (substantia nigra), n = 7–10 (ventral tegmental area). For further explanations see Figs. 1 and 2.

The regions examined in the present study and the distribution of BDNF and trkB mRNAs in the frontal brain sections are shown in Fig. 1. The 6-OHDA-induced lesion decreased the level of both BDNF and trkB mRNA in CA1, CA3 and DG of the hippocampus (lesion effect: 1) BDNF CA1 F[_1,33_] = 7.42, CA3 F[_1,28_] = 4.84, DG F[_1,34_] = 6.80; 2) trkB CA1 F[_1,35_] = 18.02, CA3 F[_1,35_] = 15.35, DG F[_1,35_] = 7.10) (Figs. 2, 3) and amygdala (lesion effect: 1) BDNF F[_1,36_] = 18.31; 2) trkB F[_1,33_] = 12.3) (Fig. 4). Additionally, the lesion decreased the BDNF (lesion effect: F[_1,30_] = 4.82) but not trkB mRNA expression in the habenula (Fig. 5) and had no influence on the nucleus accumbens (shell, core) (Fig. 6, S1 Fig), caudate-putamen (Fig. 7), substantia nigra and VTA (Fig. 8).

### The influence of chronic imipramine and pramipexole administration on the BDNF and trkB mRNA levels in brain structures

**Imipramine.** Imipramine did not influence the BDNF mRNA expression in the CA1 and CA3 but a non-significant trend (imipramine effect: F[_1,36_] = 3.51, p = 0.069) was found in the DG. The LSD post-hoc test revealed that imipramine significantly reversed the lesion-induced decreases in BDNF mRNA expression in the latter hippocampal region (Fig. 2). Moreover, imipramine decreased the level of BDNF mRNA (imipramine effect: F[_1,34_] = 5.52) in the habenula in the sham-operated rats (p<0.05, LSD post-hoc test), only (Fig. 5). In contrast, this drug did not deepen losses of the BDNF mRNA induced by the lesion in this structure (Fig. 5).

Imipramine increased BDNF mRNA (lesion x imipramine interaction: F[_1,35_] = 4.29) in the shell of the nucleus accumbens (Fig. 6), trkB mRNA in the substantia nigra (lesion x imipramine interaction: F[_1,31_] = 6.04) and VTA (lesion x imipramine interaction: F[_1,29_] = 3.87, p = 0.058), only in the sham-operated rats (p<0.05, LSD post-hoc test) (Fig. 8). No other effect of this drug on BDNF or trkB mRNA was found in the examined brain regions.

**Pramipexole.** Pramipexole influenced neither the BDNF nor trkB expression in the hippocampus (Figs. 2, 3). In contrast, pramipexole significantly decreased the BDNF mRNA level in the amygdala (pramipexole effect: F[_1,36_] = 4.59; Fig. 4), nucleus accumbens shell (pramipexole effect: F[_1,35_] = 5.57) and core (pramipexole effect: F[_1,35_] = 10.69; Fig. 6) in lesioned rats (p<0.05, LSD post-hoc test) and in the habenula (pramipexole effect: F[_1,30_] = 13.21] in the sham-operated (p<0.05, LSD post-hoc test) rats (Fig. 5). Additionally, pramipexole lowered the trkB mRNA levels in both the sham-operated and lesioned rats in the caudate-putamen (pramipexole effect: F[_1,34_] = 13.41) (Fig. 7). In the VTA, however, pramipexole increased the BDNF mRNA (pramipexole effect: F[_1,27_] = 3.92, p = 0.057) but decreased trkB mRNA levels (lesion x pramipexole interaction: F[_1,31_] = 3.88, p = 0.057) in lesioned rats (p<0.05, LSD post-hoc test), only (Fig. 8). Pramipexole influenced neither the BDNF mRNA nor trkB mRNA in the substantia nigra (Fig. 8).

## Discussion

The present study shows that bilateral 6-OHDA injections into the ventral regions of the caudate-putamen in rats reduced BDNF mRNA levels in the hippocampus, amygdala and habenula, and additionally, lowered trkB mRNA in the two former structures.

The 6-OHDA-induced damage was only moderate, as shown in our previous study (ca. 40–55% loss of dopamine in the caudate-putamen, nucleus accumbens and frontal cortex) [39]. This lesion was suggested to model depression of the preclinical stages of PD since it induced depressive-like behaviour (prolongation of immobility) in the forced swimming test but did not disturb motility of rats [39].

The present study may suggest that the aforementioned 6-OHDA-induced alterations in the BDNF and trkB expression contribute to the depressive-like behaviour in these rats. This conclusion is strongly supported by earlier studies which showed that different kinds of stress decreased BDNF and trkB expression in CA1, CA3 and/or DG of the hippocampus in animals [6,20–23,46–49]. Stress is a well-known factor triggering depressive symptoms in humans and depressive-like behaviours in animals [5–7,50]. Moreover, the significance of these hippocampal alterations for depressive symptoms was confirmed by their reversal by chronic administration of antidepressant drugs belonging to different classes [6,22,41–44,46]. Furthermore, the levels of BDNF (protein and mRNA) in the hippocampus were found to be lowered in depressed patients [10,12], and direct injections of this neurotrophin into the DG or CA3 led to antidepressant-like activity in animals [51].

In accordance to the aforementioned data, the present study also showed that a 2-week imipramine administration reversed the 6-OHDA-induced decreases in BDNF mRNA level in the DG but not in other structures. On the other hand, our previous study showed that imipramine did not influence immobility time in lesioned animals in the forced swimming test [39]. The latter result did not seem, however, to contradict the significance of hippocampal BDNF signalling for antidepressant-like effect of this drug in the 6-OHDA-induced model. This conclusion is based on the finding that imipramine induced excessive sedation in lesioned animals which may mask its therapeutic effect in the forced swimming test [39].

Since BDNF and other neurotrophins are critical regulators of the formation and plasticity of neuronal networks, decreases in BDNF signalling have been suggested to be related to the atrophy of neurons and dendrites in the hippocampus, as well as to the reduced adult neurogenesis observed in stressed animals and depressed humans [2,6,7,52]. Therefore, the present study may suggest that the lesion of dopaminergic neurons, like stress, disturbs neuronal plasticity of hippocampal circuits. This notion is in line with previous studies which showed that the 6-OHDA-induced lesion in rodents decreased the long-term potentiation and long-term depression in the DG [53,54] as well as reduced neurogenesis in the subgranular zone of the DG [55]. Similarly, the impairment of precursor cell proliferation in this region was demonstrated by Höglinger et al. [56] in MPTP-treated mice and PD patients.

Additionally, the present study showed decreases in the BDNF and trkB mRNA in the amygdala in the 6-OHDA-lesioned rats. The amygdala is a well-known structure involved in processing information underlying fear, anxiety as well as other emotional states, including depression [57,58]. The data concerning the influence of stress on neuronal plasticity of this structure in animals seemed to suggest its varying regulation in anxiety and depression. Chronic immobilization stress has been shown to induce dendritic hypertrophy in the basolateral nucleus of the amygdala [59–61], which appeared to result from an increased expression of BDNF in this structure [61,62], and was suggested to be responsible for anxiety behaviour [59–62]. On the other hand, the stress resulting from maternal deprivation [63,64], social defeat stress [65] and chronic unpredictable mild stress [25,66] reduced BDNF protein levels. The latter stress-induced alteration was reversed by antidepressant drugs along with their therapeutic effects in animal models [25,64,66]. The reduced BDNF mRNA levels in the amygdala (lateral/basolateral/basomedian complex) [67] as well as the decreased volume of this structure were also found in depressed patients [68]. Taking into account all the above data, it is tempting to suppose that, like in the hippocampus, the currently seen decreases in mRNA levels for both BDNF and its receptor trkB in the amygdala of the 6-OHDA-lesioned rats may underlie depressive-like behaviour observed by us previously [39].

We also showed that the 6-OHDA-induced lesion reduced BDNF mRNA expression in the habenula. Since this structure is small, and its BDNF (protein and mRNA) levels are low [69] (present study), we were unable to analyse its lateral and medial nuclei separately. Several recent animal and clinical studies indicated a crucial role of the lateral habenula in depression (for ref. see [70]). It has been shown that depressive symptoms in humans are related to neuronal hyperactivity of the lateral habenula, and its deep brain stimulation (DBS) produced antidepressant effects [71,72]. A potential role of BDNF signalling in the habenula in affective disorders has not been examined, yet, although the aforementioned DBS increased the serum level of this neurotrophin [73]. Since a recent study has suggested that increased neuronal activity in the lateral habenula underlies depressive-like behaviour in 6-OHDA-lesioned animals [74], the alterations of BDNF mRNA expression seen in the present study may contribute to this phenomenon.

The mechanisms responsible for a distant modulation of BDNF signalling by 6-OHDA-induced lesion may only be a matter of speculations and complex neuronal interactions may be considered. Intrastriatal 6-OHDA injections induced moderate retrograde losses of tyrosine hydroxylase-immunoreactive neurons in the substantia nigra and VTA [39]. Since the hippocampus, amygdala and lateral habenula receive inputs from the latter structure [75,76], the 6-OHDA-induced lesion of its cell bodies may reduce the number of dopaminergic terminals in these structures, as well. Moreover, the substantia nigra and VTA send projections to the midbrain raphe nuclei [75,77,78], which, in turn, innervate the hippocampus, amygdala and habenula [79]. The stimulation of serotoninergic neurons *via* 5-HT2A receptors was found to contribute to the reduction of BDNF expression in the hippocampus in stress [21,48,80]. Since the 6-OHDA-induced lesion of the nigrostriatal pathway has been found to activate the midbrain raphe nuclei in rats [81,82] and to alter serotonin transmission in the hippocampus [83], a similar regulatory mechanism on BDNF signalling in the latter structure may be expected in lesioned animals. Another possibility is related to the involvement of the lateral habenula, since it is directly innervated by the GABA/glutamatergic projections from the entopenduncular nucleus (a rodent equivalent of the globus pallidus, internal part) [84] and increased pallido-habenular connectivity has been found in 6-OHDA-induced models [85].

In contrast to distant structures, no influence of 6-OHDA injections on BDNF and trkB mRNAs was found in the lesioned regions (caudate-putamen, nucleus accumbens, substantia nigra, VTA). Both these transcripts are highly expressed by dopaminergic neurons [32,86], therefore, decreased BDNF expression along with losses of these neurons as reported earlier in lesioned animals and in PD [29,87]. However, as mentioned above, the lesion used in the present study was only partial [39], and, therefore, it might trigger compensatory enhancement of BDNF signalling in surviving dopaminergic neurons, their target non-dopaminergic neurons and glial cells which might mask its deficits resulting from the lesion. In fact, microglia and astroglia proliferation in the 6-OHDA-lesioned rodents and PD [88–90] as well as up-regulation of BDNF-immunoreactivity of glial cells surrounding fragmented nigral neurons were demonstrated [90]. The increase in trkB mRNA in striatal neurons was also observed in the above animal model [86].

Our recent results have indicated that pramipexole administered for 2 weeks reversed the depressive-like behaviour (shortened the immobility time) in the 6-OHDA-lesioned rats [39]. The present study showed that such treatment altered BDNF signalling in these animals in different brain structures. Chronic pramipexole increased BDNF mRNA in the VTA and decreased it in the nucleus accumbens (shell and core) and amygdala, lowering trkB mRNA expression in the VTA and caudate-putamen. In contrast, chronic imipramine did not influence these transcripts in the above structures in the lesioned animals.

The enhancement of BDNF mRNA expression in the VTA induced by this drug seems to be in line with its D3 receptor-specific increasing effect on this neurotrophin level in dopaminergic neurons of the primary mesencephalic culture [91]. In contrast, the mechanisms underlying its actions in the nucleus accumbens, amygdala and caudate-putamen are unknown. Besides glial cells, BDNF and trkB mRNAs in these structures are localized in non-dopaminergic neurons [27,65,69,86,92,93]. As mentioned above, all these structures (even the ventral region of the caudate-putamen) receive dopaminergic innervation from the VTA [75,76]. Therefore, their lower BDNF signalling induced by chronic pramipexole in lesioned animals might be adaptive to its enhancement in dopaminergic mesolimbic neurons. The aforementioned decreases in trkB expression in the VTA may also be adaptive.

Functional significance of the above pramipexole-induced alterations are still unclear. However, previous animal studies have indicated that an increase in BDNF signalling in the VTA-nucleus accumbens system may be related to depression. This circuitry is well-known to be engaged in motivational processes [27]. The elevated BDNF and trkB expression in dopaminergic neurons of the VTA and in medium spiny neurons of the nucleus accumbens (core and shell), together with dendritic hypertrophy of the latter neurons, were found in animals exposed to intermittent social defeat or chronic unpredictable mild stress and induced depressive-like behaviour [27,28,65]. In contrast, a decreased BDNF signalling in these structures was suggested to underlie antidepressant effect [27,94]. Therefore, our present study seems to suggest that the lowered levels of BDNF and trkB mRNAs in the nucleus accumbens (shell and core) may contribute to the pramipexole-induced antidepressant-like effects [39]. Furthermore, the reduction in trkB expression in the VTA which should restrain BDNF action on dopaminergic neurons in this structure might also be involved. This conclusion is further supported by the fact that both the aforementioned pramipexole effects, i.e. antidepressant-like action [39] and alterations in BDNF signalling in the VTA and nucleus accumbens (present study) were observed only in lesioned but not sham-operated animals.

Summing up, the present study suggests that alterations of BDNF signalling in different brain structures may underlie both the depressive-like behaviour evoked by the 6-OHDA-induced dopaminergic lesion and antidepressant-like effects of chronic pramipexole in rats. Whereas the pro-depressive effects of the lesion may be related to decreases in BDNF and trkB mRNAs expression in the hippocampus, amygdala and possibly habenula, it is the nucleus accumbens which seems to be the target for antidepressant properties of pramipexole. In contrast to pramipexole, the reversal of the lesion-induced loss of BDNF mRNA expression in the DG of the hippocampus seems to contribute to antidepressant properties of imipramine. Furthermore, it should be kept in mind that the lesion used in the present study was only moderate and, therefore, it modelled preclinical stages of PD. Future studies designed to evaluate the role of alterations in BDNF signalling in PD-associated depression and its putative significance as a biomarker of this disease are warranted.

## Supporting Information

S1 Fig The influence of 6-OHDA, pramipexole and imipramine on trkB mRNA in the nucleus accumbens. The results are expressed as the mean ± S.E.M. IMI—imipramine, LESION—lesioned rats, PRA—pramipexole, Q-B/pixel2—the mean optical density-background per area units, SHAM—sham-operated rats, SAL—saline. n = 6–8.
(TIF)
