# Supplementary figures and images for: Alterations of BDNF and trkB mRNA Expression in the 6-Hydroxydopamine-Induced Model of Preclinical Stages of Parkinson’s Disease: An Influence of Chronic Pramipexole in Rats
Source: PLoS One. 2015 Mar 4;10(3):e0117698. doi: 10.1371/journal.pone.0117698 (PMC4349741; doi:10.1371/journal.pone.0117698)

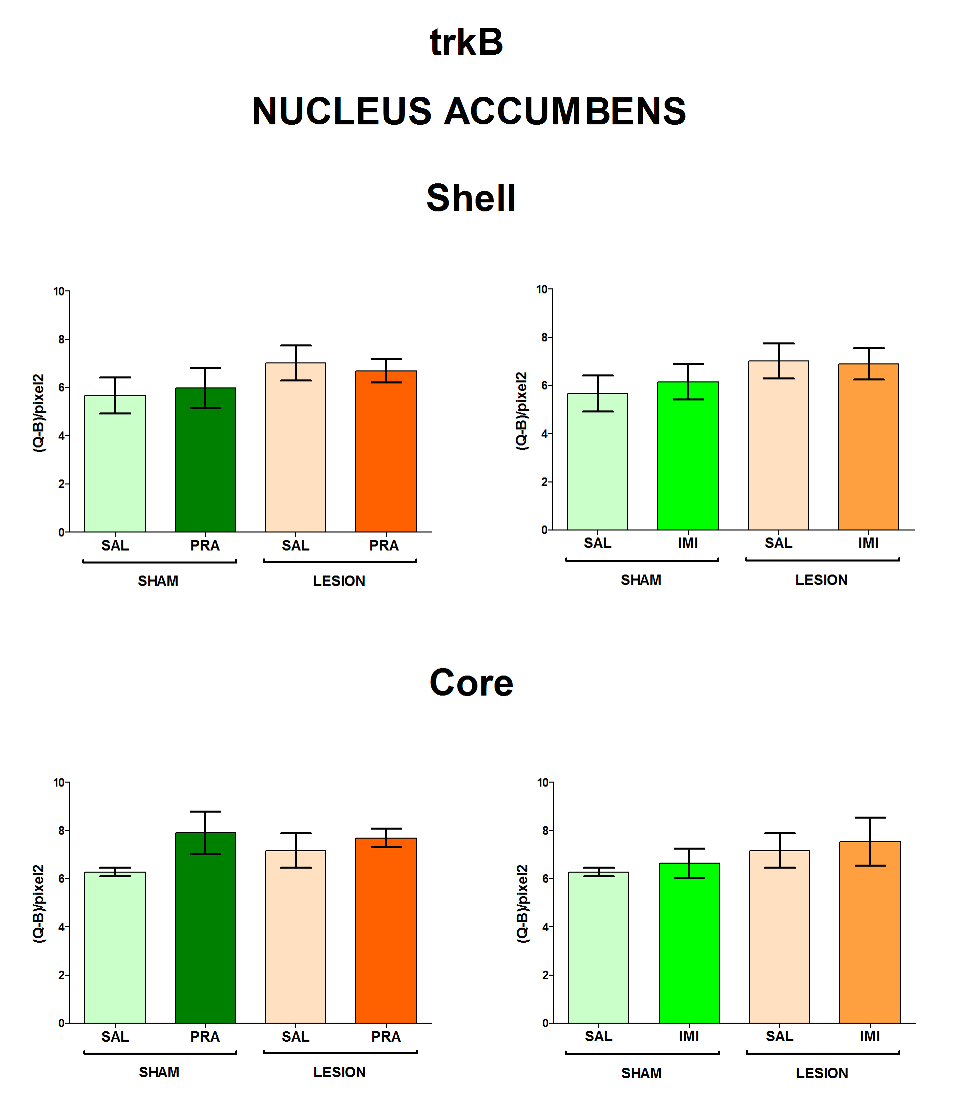

Supplement: S1 Fig — The results are expressed as the mean ± S.E.M. IMI—imipramine, LESION—lesioned rats, PRA—pramipexole, Q-B/pixel2—the mean optical density-background per area units, SHAM—sham-operated rats, SAL—saline. n = 6–8. (TIF) [file pone.0117698.s001.tif]
